# Two-dimensional MXO/MoX_2_ (M = Hf, Ti and X = S, Se) van der Waals heterostructure: a promising photovoltaic material†

**DOI:** 10.1039/d2ra03204j

**Published:** 2022-08-02

**Authors:** Aman Kassaye Sibhatu, Georgies Alene Asres, Abubeker Yimam, Tamiru Teshome

**Affiliations:** Department of Chemical Engineering, School of Chemical and Bio Engineering, Addis Ababa Institute of Technology, Addis Ababa University Addis Ababa Ethiopia abubeker.yimam@aau.edu.et +251 911950214; Center for Materials Engineering, Addis Ababa Institute of Technology, School of Multi-disciplinary Engineering Addis Ababa 1000 Ethiopia; Department of Physics, College of Natural and Social Science, Addis Ababa Science and Technology University P. O. Box 16417 Addis Ababa Ethiopia tamiruteshome@gmail.com +251 966 253 809; Department of Chemical Engineering, College Biological and Chemical Engineering, Addis Ababa Science and Technology University P. O. Box 16417 Addis Ababa Ethiopia

## Abstract

Nanoscale materials with multifunctional properties are necessary for the quick development of high-performance devices for a wide range of applications, hence theoretical research into new two-dimensional (2D) materials is encouraged. 2D materials have a distinct crystalline structure that leads to intriguing occurrences. Stacking diverse two-dimensional (2D) materials has shown to be an efficient way for producing high-performance semiconductor materials. We explored a 2D nanomaterial family, an MXO/MoX_2_ heterostructure (M = Hf, Ti and X = S, Se), for their various applications using first-principles calculations. We discovered that all of the heterostructure materials utilized are direct band gap semiconductors with band gaps ranging from 1.0 to 2.0 eV, with the exception of hexagonal HfSeO/MoSe_2_, which has a band gap of 0.525 eV. The influence of strain on the band gap of this HfSeO/MoSe_2_ material was investigated. In the visible range, we obtained promising optical responses with a high-power conversion efficiency. With fill factors of 0.5, MXO/MoX_2_ photovoltaic cells showed great PCE of up to 17.8%. The tunable electronic characteristics of these two-dimensional materials would aid in the development of energy conversion devices. According to our findings, the 2D Janus heterostructure of MXO/MoX_2_ (M = Hf, Ti and X = S, Se) material is an excellent choice for photovoltaic solar cells.

## Abstract 2

Nanoscale materials with multifunctional properties are necessary for the quick development of high-performance devices for a wide range of applications, hence theoretical research into new two-dimensional (2D) materials is encouraged.

## Graphical abstract

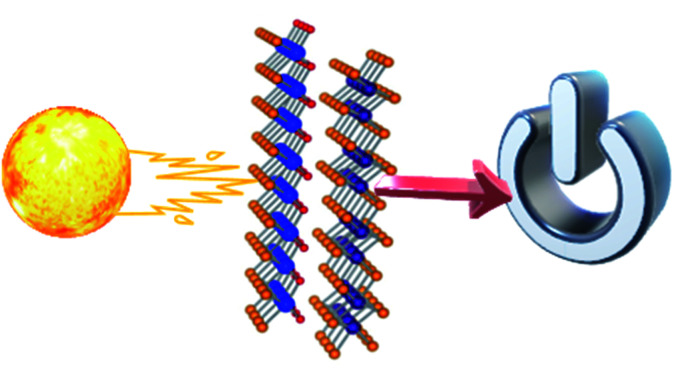

## 1 Introduction

Materials science and engineering are now more crucial than ever in everyone's lives. Solar power conversion into other kinds of energy, such as electricity (photovoltaic system) is a viable renewable energy generation approach for meeting the world's rising energy demand.^1^ Alternative energy sources with no or low greenhouse emissions are promising ways to address environmental and energy issues.^2,3^ Advanced materials are essential for energy absorption, conversion, and utilization technologies. Two-dimensional semiconductors are utilized in photocatalysts and photovoltaics, which are currently the focus of study in order to achieve a promising material for sustainable energy generation due to its innovative features,^4,5^ optically distinct and electronic properties.^6^ Despite the fact that the researcher's primary focus area is the intensified development of semiconductor materials, which still face multiple significant challenges in producing effective semiconductors, in order to address these challenges, the development of new and more efficient semiconductor materials is required.^4^ Today, the basic strategy in advanced materials science and engineering research is to tailor the materials to achieve the desired efficiencies and functionalities.^2^ For example, a Si-based photovoltaic cell has material quality and quantity disadvantages.^7^ Photovoltaic cells face cost, performance, and operating long lasting challenges.^8^ As a result, researchers are working hard to improve the performance of the PCE. Some solar cell research findings in 2D layered van der Waals heterostructures at 2% tensile strain, the PCE of MoSSe/g-GeC is 10.3% (ref. 9) SeIn_2_Te/C_2_N and TeIn_2_Se/C_2_N vdWHs have PCEs of around 12 and 16%, respectively^10^ and for AAII-Se stacking MoS_2_/MoSSe and MoS_2_/WSSe a PCE of 12.15% and 9.37%, respectively.^11^ It suggests that improving the PCE and other performance factors will take a lot of effort. To address these issues, various 2D materials need to be use with proper Janus and TMDCs that allow for the creation of favorable energy offsets that drive electron–hole separation.^12^ The following TMD monolayer materials are significant for the development of photovoltaic cells, such as MoS_2_ potential for solar cells,^11^ MoSe_2_ fast photodetection.^13^ These materials can bridge the gap owning to their superior optical, electronic, and physical properties, as well as better controllable tuning of physical dimensions. In addition, the layered structure nature of two-dimensional/two-dimensional van der Waals heterostructures have gained a lot of attention recently. They have interesting properties like tunable electronic bandgap, optical absorption, efficient charge separation and transfer, coupling effect, and low quantum confinement^12,14–17^ Janus TMDs materials, which differ from traditional 2D materials, have sparked considerable interest. Janus TMDs materials have distinct properties such as asymmetric crystal structure, intrinsic out-of-plane polarization, and piezoelectricity.^18–23^ The 2D/2D van der Waals heterostructures coupling is important, it produces a variety of interesting effects^24,25^ it is a useful method for combining various properties from 2D different materials^26^ to promote PV technology innovation.^27^ By stacking the two monolayers together, the MXO/MoX_2_ heterostructure can be constructed based on this advantage and tunable properties.^28^ According to the report, special phenomena will emerge as the two-dimensional materials layer thickness continues to decrease because it is easy to tune and control the functionalities.^29–32^ Several 2D materials have been proposed in terms of electronic and optical properties for photovoltaic cells, photodetectors, and photocatalysts.^33–35^ Two-dimensional materials have a lot of potential in PV compared to typical photovoltaic solar energy conversion materials.^36^ In general, the development of 2D heterostructures results in novel material enhancements.^10^ But the heterostructure MXO/MoX_2_ has yet to be studied. So, in order to investigate the unprecedented unique properties, we proposed hexagonal and distorted heterostructures; such as H-HfSeO/MoSe_2_, H-HfSO/MoSe_2_, H-TiSeO/MoSe_2_, H-TiSO/MoSe_2_ and T-HfSeO/MoSe_2_, T-HfSO/MoSe_2_, T-TiSeO/MoSe_2_, T-TiSO/MoSe_2_ stack to investigate the potential of optical properties for PV application.^15,23,37^ We investigated the electronic and optical properties of the materials in a systematic manner. Janus (MXO) and TMDs (MoX_2_) are the general formulas for the crystal structure, where M is a group IV atom (Zr, Ti) and X is a chalcogen (S, Se). Valance electron configuration of hafnium (4f^14^ 5d^2^ 6s^2^), titanium (3d^2^ 4s^2^), molybdenum (4d^5^ 5s^1^), sulfur (3s^2^ 3p^4^), selenium (3d^10^ 4s^2^ 4p^4^), and oxygen (2s^2^ 2p^4^).

## 2 Computational methods

Our calculations were carried out using the Vienna *ab initio* simulation package (VASP) based on density functional theory (DFT) with the projector augmented-wave method.^22,38–40^ A general gradient approximation of Perdew, Burke, and Ernzerhof (PBE).^32^ To account for the PBE functional's underestimated band gap, we used the hybrid density functional Heyd–Scuseria–Ernzerhof (HSE06).^41^ Vacuum space was set to 25 Å along the *c*-axis to differentiate the interactions of the adjacent layer. The Brillouin zone was sampled using the Monkhorst–Pack scheme, and a mesh is adopted of 9 × 9 × 1 and 15 × 15 × 1 the wave function expansion of the kinetic energy cutoff set to 520 eV. The convergence threshold criterion is less than 0.01 eV Å^−1^ for ionic relaxation and set for the energy up to 10^−5^ eV. Optical absorption characteristics were investigated by setting up a *k* grid 15 × 15 × 1. The optical properties calculations for all heterostructures are based on the frequency-dependent dielectric function.^18^ The binding energy between the two monolayers is used to calculate the hetero-structure stability, based on the equation which is defined as^42^ expressed in eqn (1) below.

1 *E*_b_ = *E*_MXO/MoX_2__ − *E*_MXO_ − *E*_MoX_2__

where, *E*_b_ is the binding energy, *E*_MXO_, *E*_MoX_2__ and *E*_MXO/MoX_2__ total energy of the mono-layers: Janus, TMDs and heterostructure, respectively. The optical properties were calculated using the time-dependent (TD) DFT scheme to include electron–hole interactions^43^ in the random phase approximation (RPA) approach, which includes local field effects at the Hartree level. Only transitions between bands are considered. For a transitionally invariant system, the Fourier transform of the frequency-dependent symmetric dielectric matrix in the RPA is given by.^44^2
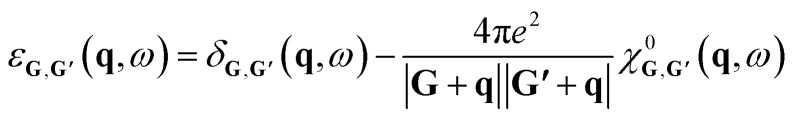
where **G** and **G′** are reciprocal lattice vectors and **q** stands for the Bloch vector of the incident wave. The matrix *χ*^0^(**q**, *ω*) is the irreducible polarizability matrix in the case of the independent particle derived by Adler and Wiser^45,46^ in the context of the self-consistent field approach. Optical properties of any system are in general calculated with the help of a frequency dependent complex dielectric function *ε*(*ω*) = *ε*_1_(*ω*) + i*ε*_2_(*ω*). The imaginary part is determined by a summation over empty states using the equation:
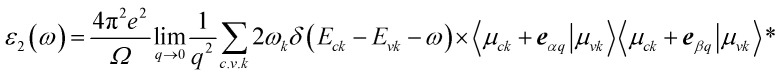
where *ω*, the frequency of electromagnetic (EM) radiation in energy unit is *Ω* represents the volume of the primitive cell, *q* is the electron momentum operator, *c* and *v* are the conduction band and valence state, respectively, *ω*_*k*_, is the *k* point weight *E*_*ck*_, *E*_*vk*_ and *μ*_*ck*_, *μ*_*vk*_ are the eigenvalues and wave function at the *k* point, respectively and ***e***_*α*_, ***e***_*β*_ are unit vectors for the three Cartesian direction.

## 3 Result and discussion

### 3.1 Structural and energetic stabilities

**Fig. 1:**
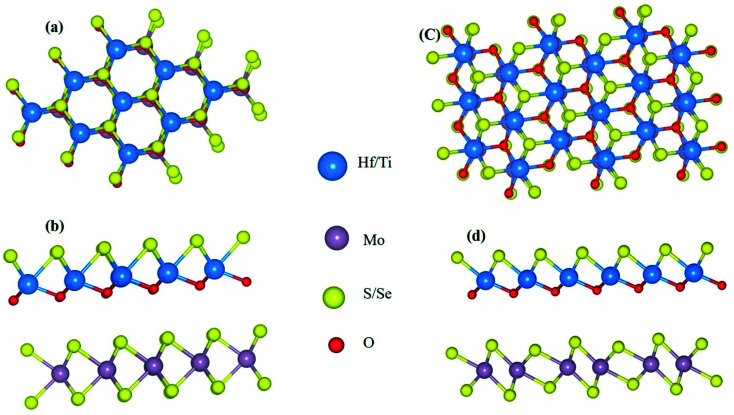
Diagram geometrical structure (a) top and (b) side views of MXO/MoX_2_ (M = Ti or Hf; X = S or Se) heterostructure in 2H phase; (c) top and (d) side views of MXO/MoX_2_ in 2T phase. M (M = Ti or Hf), Mo, X (S or Se), and O are represented by the blue, grey, yellow, and red spheres, respectively.

2D Janus MXO/MoX_2_ heterostructures (M = Ti, Hf; X = S or Se) are derivatives of their TMD MX_2_ counterparts (M = Ti, Hf; X = S or Se). MXO monolayers, like MX_2_, are composed of three atomic layers stacked in the sequence X–M–O, with the crystal structure arranged in two different ways.^16,47^ Hexagonal and distorted the ESI Fig. S1† shows a top and side view of the hexagonal and distorted Janus and TMDs monolayer with its heterostructure. To build the most equilibrium configuration of the hexagonal and distorted MXO/MoX_2_ hetero-structure, individual monolayers are initially investigated. The calculated unit cell lattice constants and band gap of HfSeO, HfSO, TiSeO, TiSO, MoSe_2_ and MoS_2_ are 3.4 Å & 1.3 eV, 3.32 Å & 2.67 eV, 3.15 Å & 1.2 eV, 3.1 Å & 1.6 eV, 3.3 Å & 1.5 eV and 3.2 Å & 1.53 eV respectively, the monolayer's sample band structure and PDOS in the ESI data Fig. S2 and S3,† the obtained results coincide with the earlier findings (Fig. 1).^16,47^

We checked various stacking configurations of the heterostructure to explore the one with the highest stability. Hence, the phase structure of 2D materials governs the physical and chemical properties.^23^ By construct the possible stacking configurations for 2H-phase 1 × 1 unit cell stacking AA, A_1_A, AA_1_ and for 2T-phase 2 × 2 MXO and MoX_2_ monolayers were stacked on top of one another to create the distorted MXO/MoX_2_ heterostructures A_1_, A_2_, A_3_ & A_4_ stacking. Based on this configuration A_1_A from the 2H-phase and A_2_ from 2T the phase has the lowest binding energy which is the more stable one in comparison to the other stacking and selected. The binding energy was found to be negative, indicating that the formation of the MXO/MoX_2_ heterostructure configurations are energetically favourable.^38,48^ The calculated heterostructure binding energy is close to the theoretically predicted value and, in some cases, more stable than the other typical vdW heterostructures listed as; MoS_2_/WSSe, MoS_2_/MoSTe, & MoS_2_/MoSSe is −13, −3 and −9 meV respectively.^11^ Experimentally measured binding energies of MoS_2_/MoSe_2_ is in between −100 meV to −200 meV (ref. 49) graphene/WSe_2_ is −55.92 meV,^50^ GaN/Bas is −57 meV,^51^ graphene/MoS_2_ is −58.1,^52^ Tables S1, S2 and Fig. S4 in the ESI section† illustrate the various stacking binding energy, band gap, and PDOS results. The negative energies indicate the structural stability of the materials. As a result, the most stable configurations of heterostructures are considered in the following discussion.

One of the criteria for classifying a material to form a van der Waals heterostructure is the interlayer distance, better to be in the range of 3–4 Å.^53^ The interlayer distance of the van der Waals heterostructure is typical van der Waal equilibrium spacing within the acceptable range, as shown in Table 1. The lattice mismatch is the other important parameter to take into account when considering a material as a van der Waals heterostructure.^54^ The two-dimensional monolayer is easily transferred onto a two-dimensional crystal substrate to form a van der Waals heterostructure that allows the combination of lattice mismatched materials.^55^ The lattice matching ratio between two individual monolayers is good to be less than 5%.^56,57^ The structural match of crystals can be measured using the lattice mismatch, expressed as 
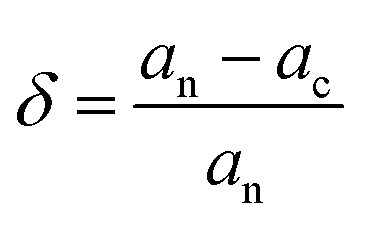
 where *a*_n_ and *a*_c_ are the lattice constants of the optimized Janus and TMDs monolayer respectively.^58^ The calculated value of the lattice mismatch of the constructed MXO/MX_2_ vdW heterostructure is in Table 1 below indicates suitable calculation results all results are less than 5% which concise with other lattice mismatch of van der Waals heterostructure such as; MoS_2_/CdS is 3.92%,^59^ SrS/SrSe is 3.91%,^56^ GaN/BAs is 4.53%,^57^ MoS_2_/MoSTe is (5.059%)^11^ so that it is possible to form a van der Waal heterostructure. Based on the aforementioned parameters, we determined that the material is a vdW heterostructure.

**Table 1:** The calculated heterostructure lattice mismatch (*Δ*), interlayer distance (*d*), binding energy (*E*_b_) and bandgap (*E*_g_)

Janus/TMDs heterostructures	*Δ* (%)	*d* (Å)	*E*_b_ (eV)	*E*_g_ (eV)	
				PBE	HSE06
H-HfSeO/MoSe_2_	2.94	3.819	−0.178	1.396	1.655
H-TiSeO/MoSe_2_	4.54	3.834	−0.234	1.443	2.202
H-HfSO/MoS_2_	3.61	3.859	−0.093	1.669	2.102
H-TiSO/MoS_2_	3.11	3.836	−0.139	1.972	2.701
T-HfSeO/MoSe_2_	4.14	4.005	−0.194	0.525	0.714
T-TiSeO/MoSe_2_	4.24	4.002	−0.149	1.014	1.303
T-HfSO/MoS_2_	4.18	3.980	−0.114	1.056	1.205
T-TiSO/MoS_2_	4.29	3.940	−0.113	1.464	1.824

It is worth noting that the atomic bond lengths *d*_M–O_ and *d*_M–X_ are inequitable due to electronegativity and atomic size. For example, *d*_Ti–Se_ = 2.7048 Å which is larger than *d*_Ti–O_ = 2.1092 Å. Regarding the Janus structure the distinction between the top and bottom atoms leads to the charge being more transferred *d*_Ti–O_ than from *d*_Ti–Se_. The calculated heterostructure is shown in Table 1. Bandgap, binding energy, lattice mismatch, and Interlayer distance are all variables to consider.

### 3.2 Electronic structures

**Fig. 2:**
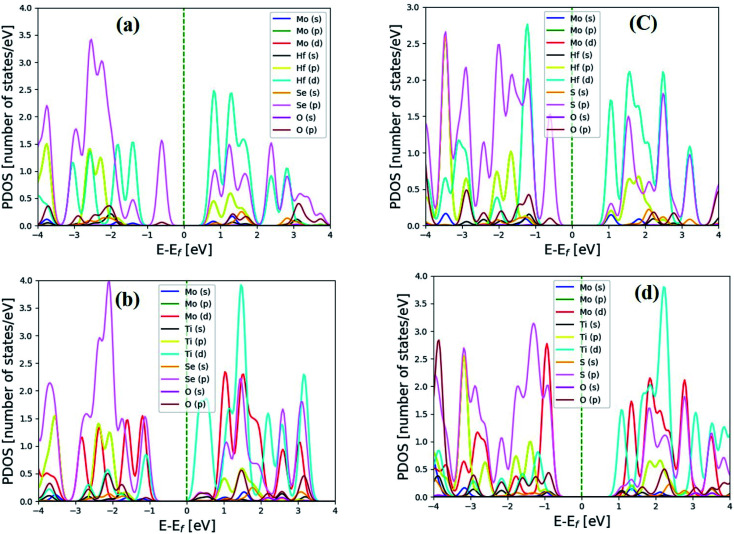
Electronic projected density of state (PDOS) for 2H heterostructure of (a) HfSeO/MoSe_2_, (b) TiSeO/MoSe_2_, (c) HfSO/MoSe_2_, and (d) TiSO/MoSe_2_. The Fermi level has been set to a value of zero.

**Fig. 3:**
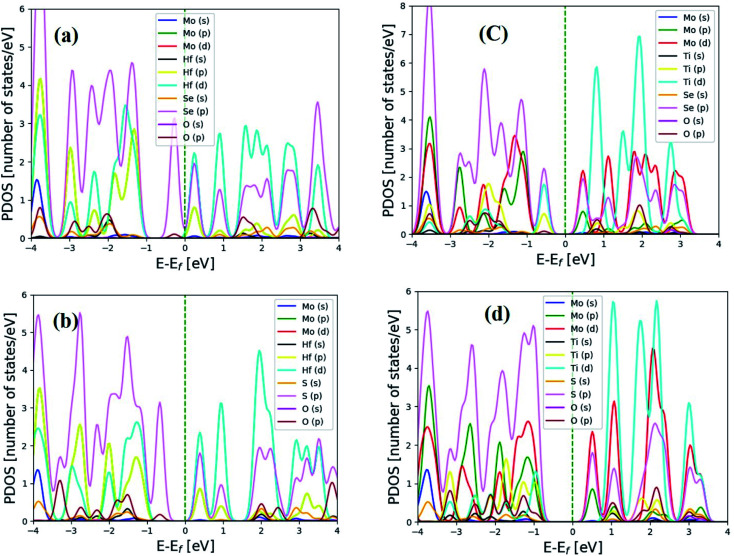
Electronic projected density of state (PDOS) for 2T-phase (a) HfSeO/MoSe_2_, (b) HfSO/MoS_2_, (c) TiSeO/MoSe_2_, and (d) TiSO/MoS_2_ heterostructure. The Fermi level has been set to zero.

To determine the characteristics of electrons that the individual atom contributions to the chosen hexagonal and distorted structure, the orbital projected density of states (PDOSs) was computed. Fig. 2(a–d) and 3(a–d) depict the heterostructure's 2H and 2T phases, respectively. From the PDOS conduction band maximum is dominated by the 2s orbital of the O atom above the Fermi and is mainly contributed by the 5d orbitals of Hafnium atoms below the Fermi level and also above and below the Fermi level in the 2T-phase heterostructure, 4p and 2s orbitals dominated, respectively. As shown in Fig. 2(a), below the Fermi level, the 5d orbital of HfSeO/MoSe_2_ is the main contributor to the valence band, whereas the 4p of Se and 2s oxygen atoms contribute to the conduction band. There is a large shift of the conduction bands towards the Fermi level for TiSeO/MoSe_2_, as seen in Fig. 2(b), and there is also an overlap between the O (d) orbitals and the Ti (d) orbitals. Fig. 3(d) shows a similar picture for the TiSO/MoS_2_ system. Due to the electron transfer is crucial for the PDOS's apparent characteristics. Fig. 3(a–d) depicts distorted heterostructure to demonstrate that the MXO contributes not only to the lowest CB but to the highest VB where the highest VB are commonly formed by s-electrons and the bottom-most CB is formed by d-electrons.

The MXO layers gave rise to the VBM and CBM. The PDOS findings suggest that building an MXO and MoX_2_ based heterostructure is advantageous for energy-related semiconductor materials. All monolayers of the Janus material are indirect band gap,^16,47^ whereas the heterostructure MXO/MoX_2_ are all obvious direct band gap semiconductors. The band gap values that were calculated for the heterostructure are listed in Table 1, except for the distorted HfSeO/MoSe_2_ heterostructure, all the calculated band gap values are in the range of between 1–2 eV, indicating that the values are suitable for photovoltaic applications.^40^ For the case of we HfSeO/MoSe_2_ investigated this material's strain effect on the bandgap shown Fig. S5 and S6 in the ESI.† The Heyd–Scuseria–Ernzerhof hybrid functional (HSE06) is also used to obtain more accurate band gap values. We investigated the power conversion efficiency of the heterostructures below for further analysis.

### 3.3 Optical properties of the heterostructure

**Fig. 4:**
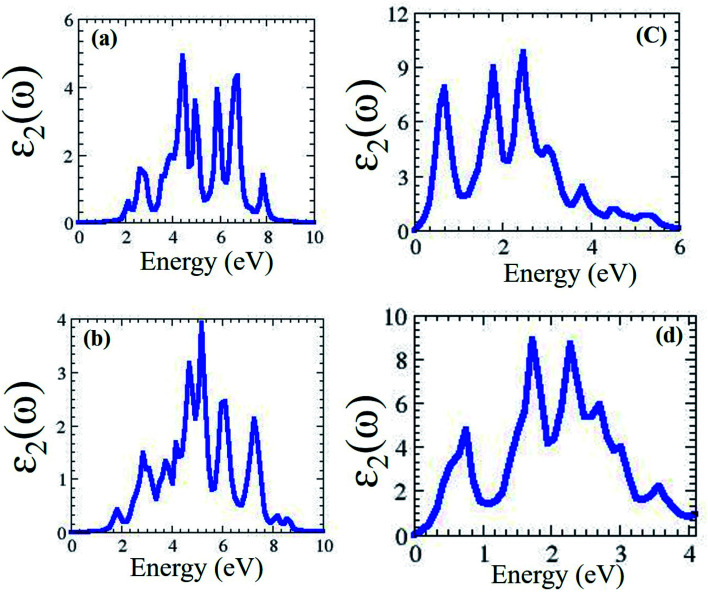
2H phase optical properties for (a) HfSeO/MoSe_2_, (b) HfSO/MoS_2_, (c) TiSeO/MoSe_2_, and (d) TiSO/MoS_2_ heterostructure.

In the optical spectra, one can expect only π to π* and σ to σ* transitions as allowed if the light is polarized parallel to the heterostructure of Janus MXO/MoX_2_. In contrast, only π to σ* and σ to π* transitions are allowed if the light is polarized perpendicular to the heterostructure. One of the most significant criteria to consider when constructing photovoltaic devices is the optical absorption properties of MXO/MoX_2_ heterostructures. We were computed and described the frequency dependent imaginary part of the dielectric function *ε*_2_(*ω*) hence it is inextricably linked to the electronic band structure of the material. The complex dielectric function *ε*(*ω*) = *ε*_1_(*ω*) + i*ε*_2_(*ω*), where *ε*_2_(*ω*) and *ε*_1_(*ω*) are the imaginary and real parts of this dielectric function. The absorption spectrum as shown in Fig. 4(a–d) hexagonal of MXO/MoX_2_ heterostructures.

**Fig. 5:**
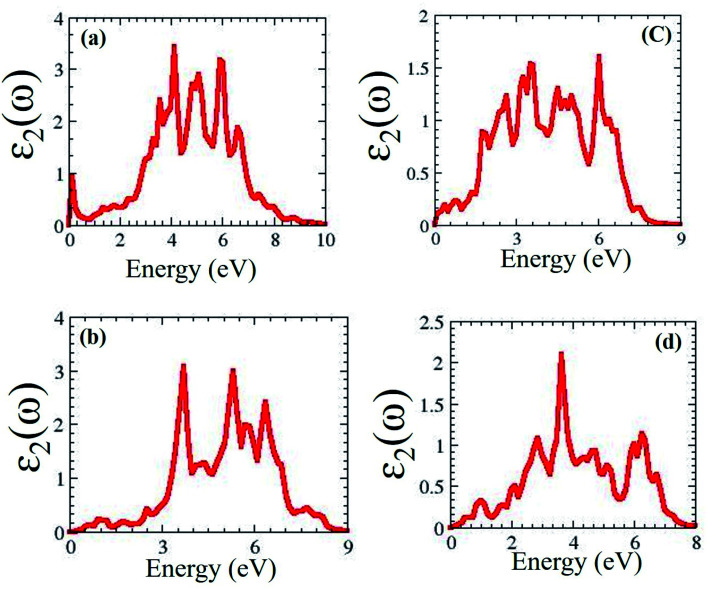
Optical properties of 2T-phases (a) HfSeO/MoSe_2_, (b) HfSO/MoSe_2_, (c) TiSeO/MoSe_2_, and (d) TiSO/MoS_2_.

We predicted that MXO/MoX_2_ has absorption peaks in the visible region, with contributions from the O (s) and Hf/Ti (d) orbitals. For the 2T-phase of HfSeO/MoSe_2_, HfSO/MoS_2_, TiSeO/MoSe_2_, and TiSO/MoS_2_, the notable maximum peaks are 4.4 eV, 3.4 eV, 5.3 eV, and 2.1 eV, respectively. Fig. 5(a–d) shows the dielectric function *ε*_2_(*ω*) of distorted MXO/MoX_2_ vdWs heterostructures in the visible/UV spectral region of 0–6.2 eV.

As a result, the maximum optical absorption peak for the 2T phase of HfSeO/MoSe_2_, HfSO/MoS_2_, TiSeO/MoSe_2_, and TiSO/MoS_2_ was seen at 4.2 eV, 3.4 eV, 3.5 eV, and 4.0 eV, respectively. One of the most defining characteristics of 2D semiconductors is their synergistic relationship with light. The photovoltaic should absorb visible light to maximize energy conversion. The calculated value of the visible light-absorbing semiconductor material. In general, heterostructures have a high dielectric constant, and the dielectric function value at zero energy is critical for photovoltaic applications. Which are very similar to the most promising material in photovoltaic cells.

### 3.4 Solar photovoltaic cells

van der Waals heterostructures with reduced layer thicknesses are a promising new development technology for ultrathin, small, and light PV solar energy.^60,61^ A suitable band gap is required for the stable MXO/MoX_2_ heterostructure, which is important for photovoltaic cells.^62^ We obtained a band-gap range that have a strong absorbance in the visible range. The most commonly used parameter to assess the performance of solar cell in power conversion efficiency (PCE).^63^ As a result, the effectiveness of all these heterostructures in photovoltaic applications is assessed based on the PCE value calculated. The efficiency of a solar cell is defined as the ratio of energy output to input energy of the sun.^64^ Using^65^ method, we computed the power conversion efficiency (PCE) *η* of the MXO/MoX_2_ vdW heterostructure. To compute the maximum PCE the most important indicator for describing a photovoltaic device is with the following equation used to calculate the PCE, which is articulated below.3
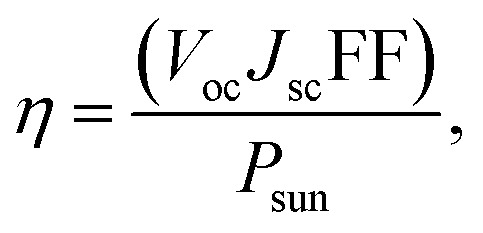
where, the open circuit voltage is denoted by *V*_oc_ (mV), *J*_sc_ (mA cm^−2^) represent short circuit-current, FF is the fill factor and the total incident solar radiation is denoted by *P*_solar_ (W m^−2^).

4 *eV*_oc_ = *E*_g_ − *E*_loss_,

where, *E*_g_ is band gap, *J*_sc_ denotes the maximum short-circuit current density and calculated as shown, and *S*(*E*) is calculated using the NREL AM1.5 dataset.5
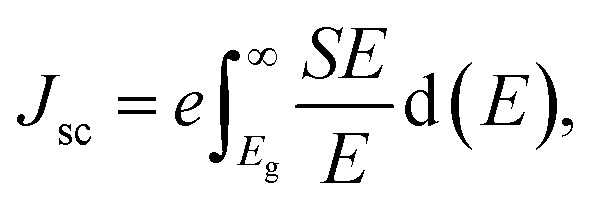


*P*_sun_ is calculated using eqn (5):6
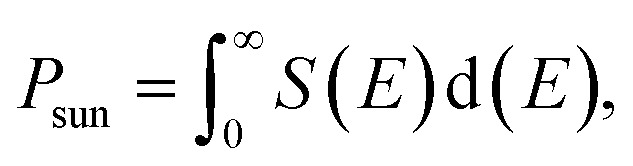


In 2D heterostructure solar cell structures, fill factors are commonly in the 0.3–0.5 range,^66^ so we considered this value. We use a value of 0.3 for energy loss (*E*_loss_).^66,67^

The PCE values that were computed are shown in Table 2 above. The calculated PCE with respect to band gap can be divided in to two groups: the optimum PCE, of which five of the materials are nearly in the optimal range, obtained at a band gap of 1 < *E*_g_ < 1.45 eV, which is between 10 to 18 percent for a fill factor 3 to 5 respectively. As a result, we conclude that MXO/MoX_2_ heterostructure material with direct band gap values it is suitable for photovoltaic cell1 with a very good efficiency. The PCE for the structures which have a band gap of *E*_g_ < 1 eV & *E*_g_ > 1.45 eV are between 5–10% and 7–15% respectively. Because the PBE functional underestimates the band gap, we used the hybrid density functional Heyd–Scuseria–Ernzerhof (HSE06) to obtain more accurate results. Table S3, Fig. S7 and S8† provide additional information on the obtained results. In terms of PCE, the material studied, it is significantly superior than the following heterostructure materials FF 0.57 MoS_2_/WSSe (9.37%),^11^ with fill factor of 0.31 WSe_2_/MoS_2_ (4.32%),^68^ for FF 57% MoS_2_/P-Si (5.23%).^69^

**Table 2:** Shows the calculated efficiency of photovoltaic conversion

Janus/TMDs	Band gap (eV)	Band gap (eV)	PCE (%)
	HSE06	PBE	
H-HfSeO/MoSe_2_	1.655	1.396	10.68–17.80
H-TiSeO/MoSe_2_	2.202	1.443	10.45–17.42
H-HfSO/MoS_2_	2.102	1.669	9.26–15.43
H-TiSO/MoS_2_	2.701	1.972	7.17–11.95
T-HfSeO/MoSe_2_	0.714	0.525	4.57–7.61
T-TiSeO/MoSe_2_	1.303	1.014	10.19–16.98
T-HfSO/MoS_2_	1.205	1.056	10.30–17.17
T-TiSO/MoS_2_	1.824	1.464	10.34–17.23

## 4 Conclusion

The structural, electrical, and optical properties of MXO/MoX_2_ hexagonal and distorted heterostructure materials were investigated. All of these compounds are semiconductors. MXO/MoX_2_ semiconductors absorb more visible light and gather photons more efficiently from visible to ultraviolet wavelengths. This implies that these materials have a high solar energy absorption efficiency and, as a result, a higher photovoltaic efficiency. This provides the best opportunity to create new materials. It should be mentioned that the two basic criteria for solar cell materials are: with a band gap ranging from 1.0 eV to 2.0 eV and having a high optical absorption. The photovoltaic activities of the hetero structure are superior due to the very effective charge separation caused by the direct band nature, as well as the high optical absorption coefficient in the visible area. The calculated Power conversion efficiency values computed indicate best efficient solar cells for next-generation energy harvesting applications. As a result, it was observed that MXO/MoX_2_ vdW heterostructures were discovered to have excellent electrical and optical properties, as well as a high photovoltaic conversion efficiency, making them attractive for use in solar cells.

## Conflicts of interest

There are no conflicts to declare.

## Supplementary Material

RA-012-D2RA03204J-s001
