# Supplementary material for: Two-dimensional MXO/MoX2 (M = Hf, Ti and X = S, Se) van der Waals heterostructure: a promising photovoltaic material
Source: RSC Adv. 2022 Aug 2;12(33):21270–9. doi: 10.1039/d2ra03204j (PMC9344374; doi:10.1039/d2ra03204j)
Supplement: RA-012-D2RA03204J-s001 [file RA-012-D2RA03204J-s001.pdf]

Electronic Supplementary Material (ESI) for RSC Advances.
This journal is © The Royal Society of Chemistry 2022

Supporting Information

Two-Dimensional MXO/MoX_2 (M=Hf, Ti and X= S, Se) Van der Waals Heterostructure: A Promising Photovoltaic Material

Aman kassaye Sibhatu[1,4], Georgies Alene,[2] Abubeker Yimam[1*], Tamiru Teshome[3]*

[1] Department of Chemical Engineering, School of Chemical and Bio Engineering, Addis Ababa institute of Technology, Addis Ababa University, Addis Ababa, Ethiopia.

[2]Center for Materials Engineering, Addis Ababa Institute of Technology, School of Multi-disciplinary Engineering, Addis Ababa, 1000, Ethiopia

[3] Department of Physics, College of Natural and Social Science, Addis Ababa Science and Technology University, P. O. Box 16417, Addis Ababa, Ethiopia.

[4] Department of Chemical Engineering, College Biological and Chemical Engineering, Addis Ababa Science and Technology University, P. O. Box 16417, Addis Ababa, Ethiopia.

*Corresponding authors; email: - tamiruteshome@gmail.com; tel:- +251 966 253 809
abubeker.yimam@aau.edu.et; tel:- +251911950214

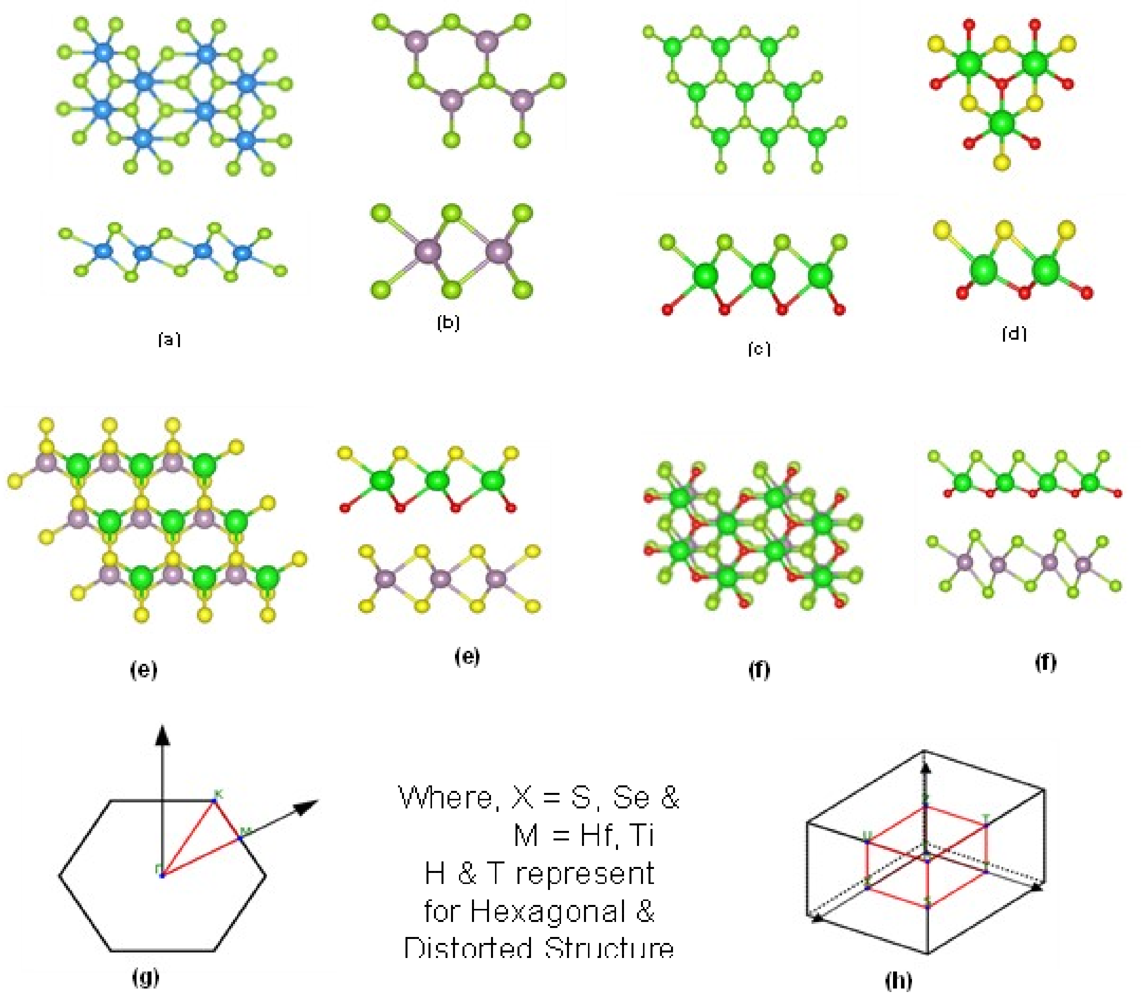

Figure S1: Top and side perspectives Janus and TMDs monolayers with heterostructure (a) T-MoX2, (b) H-MoX2, (c) H-MXO, (d) T-MXO, (e) H-MXO/MoX2, (f) T-MXO/MoX2, (g) 2H phase Brillion zone (GMKG), and (h) T phase Brillion zone (GXSYG)

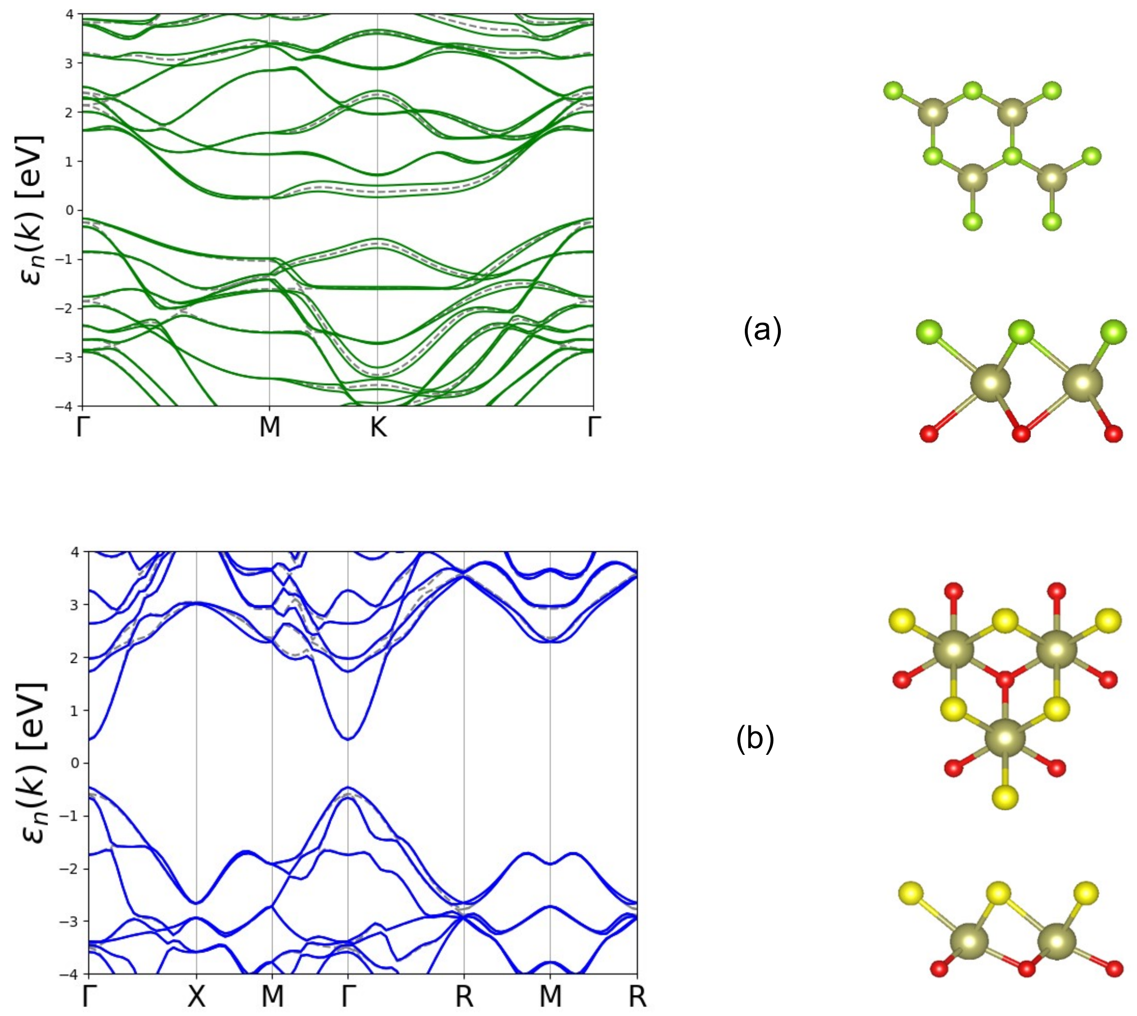

Figure S2: Band structure of Janus with crystal structure (a) hexagonal HfSeO and (b)distorted HfSO

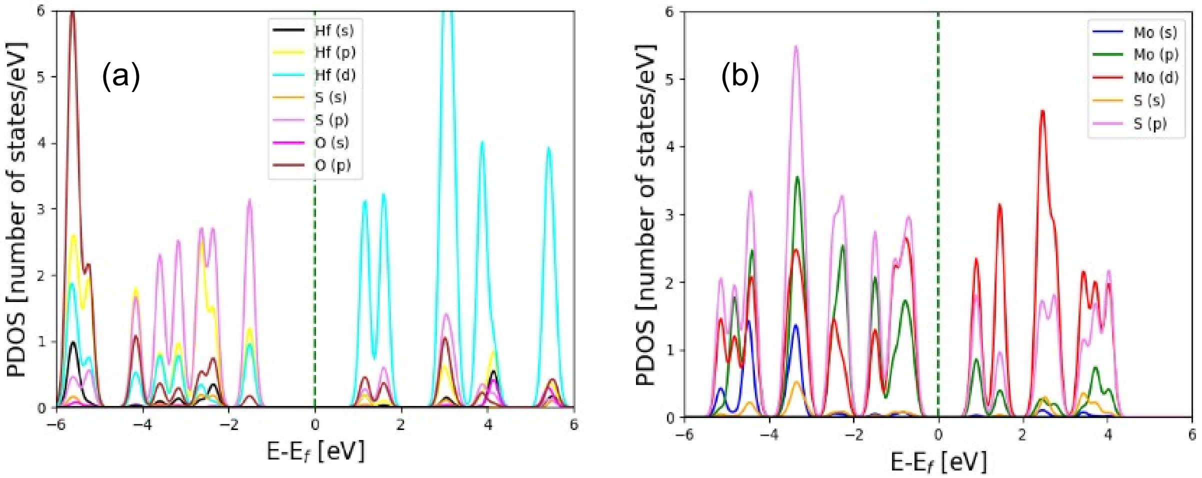

Figure S3: PDOS of Distorted (a) HfSO and (b) MoS2

Table S1: The compared hexagonal stacking Total (Et), Janus (EJ), TMDs (ET), and binding (Eb) energy

	heterostructure	Energy (eV)	Binding Energy (eV)	
Hexagonal (H) Stack	AA	Et=-39.042526	E_b=-0.01043	@ k=15x15x1 Ecut=520ev a=b=3.35Å d=3.5Å
		$E_{J=}$-21.007891		
		E_T=-18.02420		
	A1A	$E_{t\,=}$-39.578522	E_b=-0.176152	
		$E_{J=}$-19.112645		
		$E_{T\,=}$-20.289725		
	AA1	$E_{t\,=}$-39.541914	E_b=-0.140135	
		$E_{J\,=}$-19.112066		
		$E_{T\,=}$-20.289713		

Table S2: Total energy and band gap of the distorted (T) stacking are shown were, A1, A2, A3 & A4 for distorted (T) stacking

Stacking	A_1	A_2	A_3	A_4
Energy (eV)	-90.679	-90.679	-90.773	-90.64
Band Gap (eV)	1.158	1.212	1.00	1.09

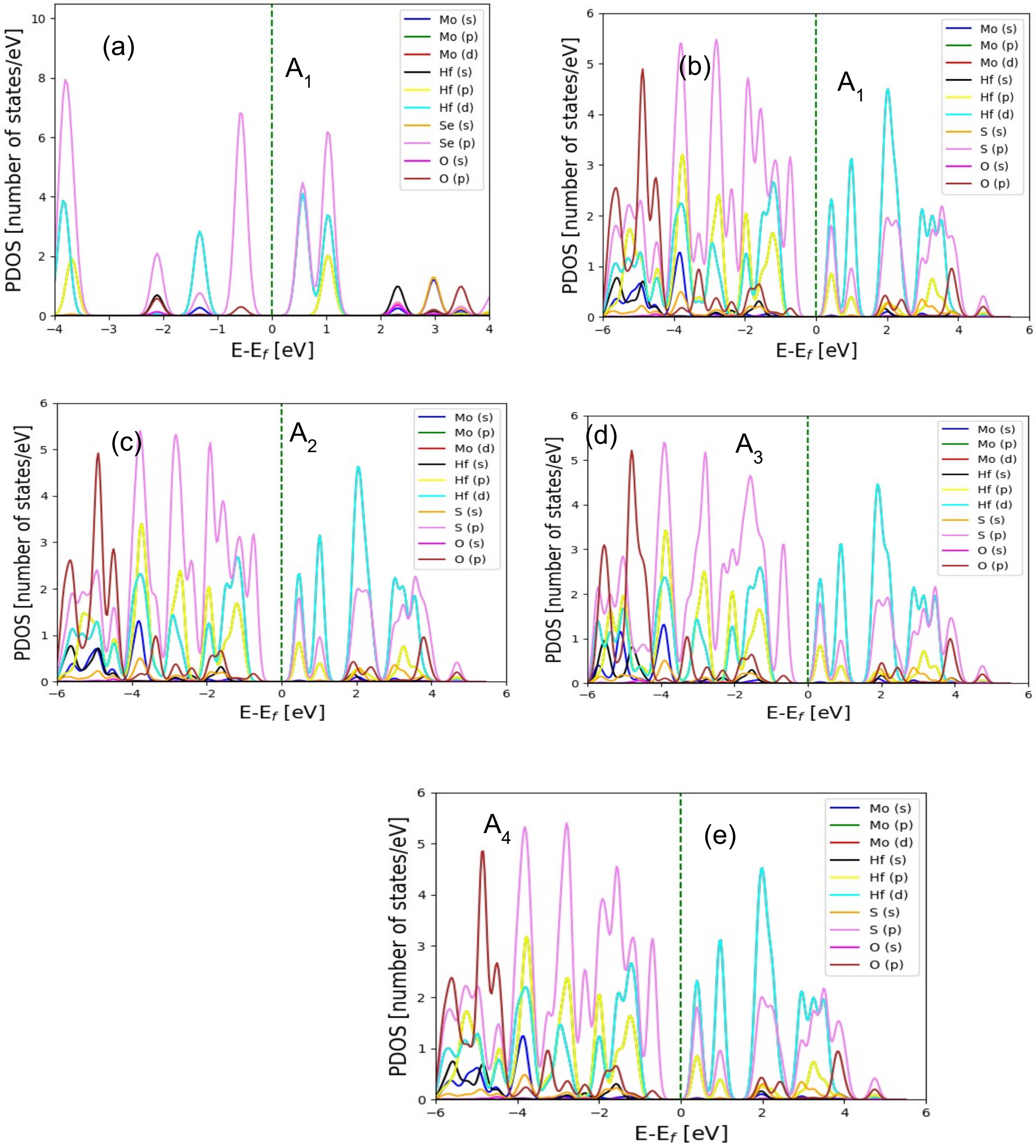

Figure S4: PDOS of the selected (a) A1A hexagonal Stack and distorted (b) A1, (c) A2, (d) A3, and (e) A4 stacking

Strain effects on band gap

The strain effects are investigated here. The strain considerably changes the band structure of the material HfSeO/$MoSe_2$, which has a band gap of 0.52eV, which is among the researched materials with a tiny band gap. We found as shown in the Figure-1 below that the band gab change when applying a strain (-6% to +6%) on hetero-structure during compressive strain it increased while the tensile reduced the band gap. The band gap become maximum is 1.2eV at the compressive strain of -6%. As a result, we can tune the band gap using strain engineering, and in our case, the band gap increases during compression.

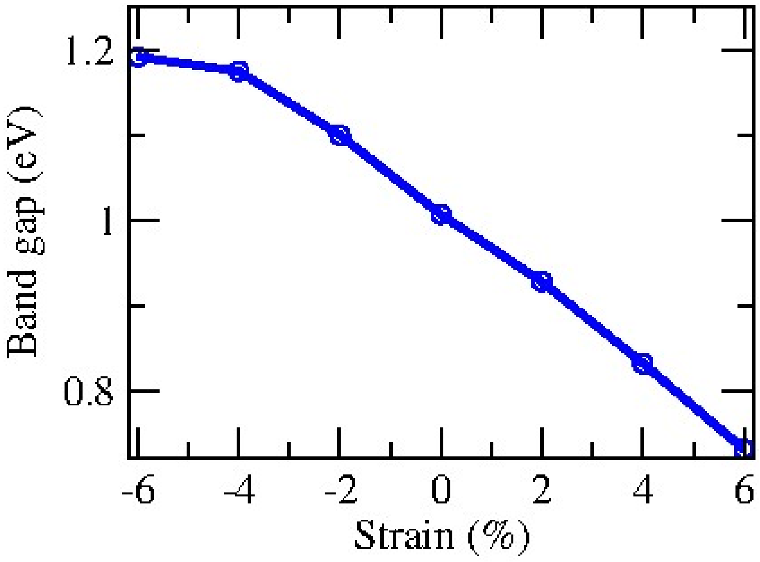

Figure S5: Strain vs band gap for HfSeO/$MoSe_2$

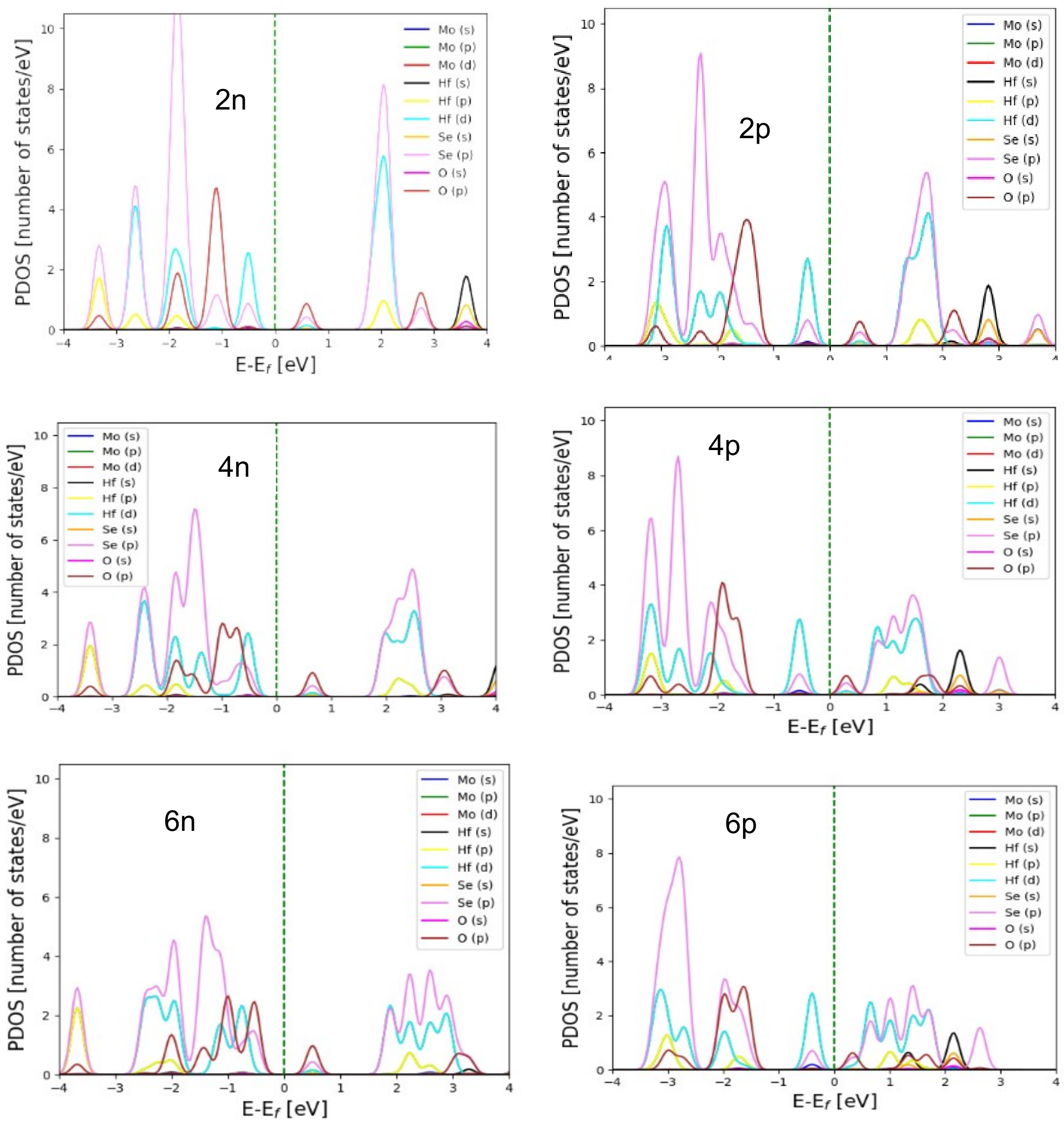

Figure S6: depicts the change in PDOS for the specified strain to HfSeO/$MoSe_2$ (compressive strain increments of -2%, -4%, and 6%, and tensile strain increases of +2%, +4%, and +6%). Where, n represents compressive strain and p represents tensile strain.

Table S3 Bandgap values of the heterostructure using PBE and HSE06 functionals

Heterostructure	Bandgap (eV)	
	PBE	HSE06
H-HfSeO/$MoSe_2$	1.396	1.925
H-TiSeO/$MoSe_2$	1.443	2.302
H-HfSO/MoS_2	1.669	2.203
H-TiSO/MoS_2	1.972	2.801
T-HfSeO/$MoSe_2$	0.525	0.725
T-TiSeO/$MoSe_2$	1.014	1.513
T-HfSO/MoS_2	1.056	1.642
T-TiSO/MoS_2	1.464	1.824

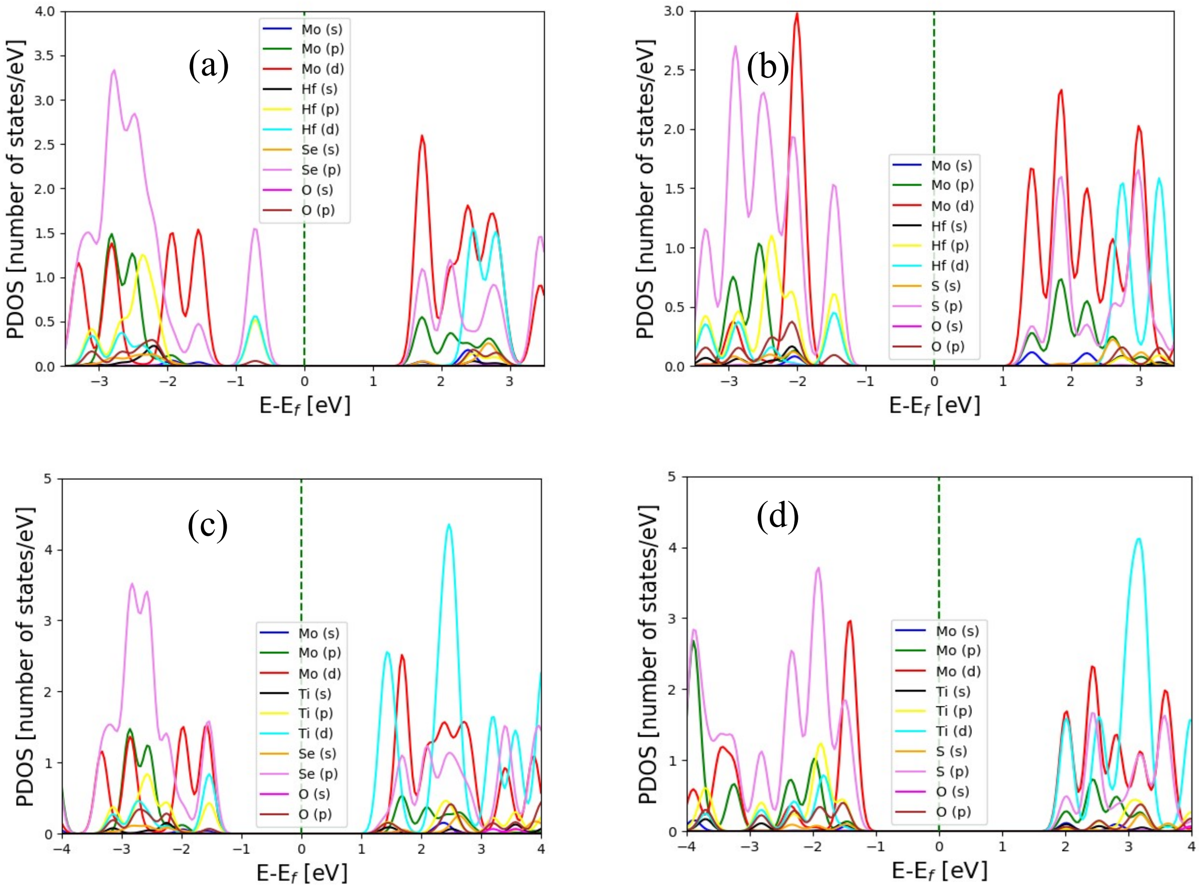

Figure S7: Bandgap using hybrid functional for Hexagonal heterostructure (a)H-HfSeO/MoSe2, (b)H-HfSO/MoS2, (c)H-TiSeO/MoSe2, and (d)H-TiSO/MoS2

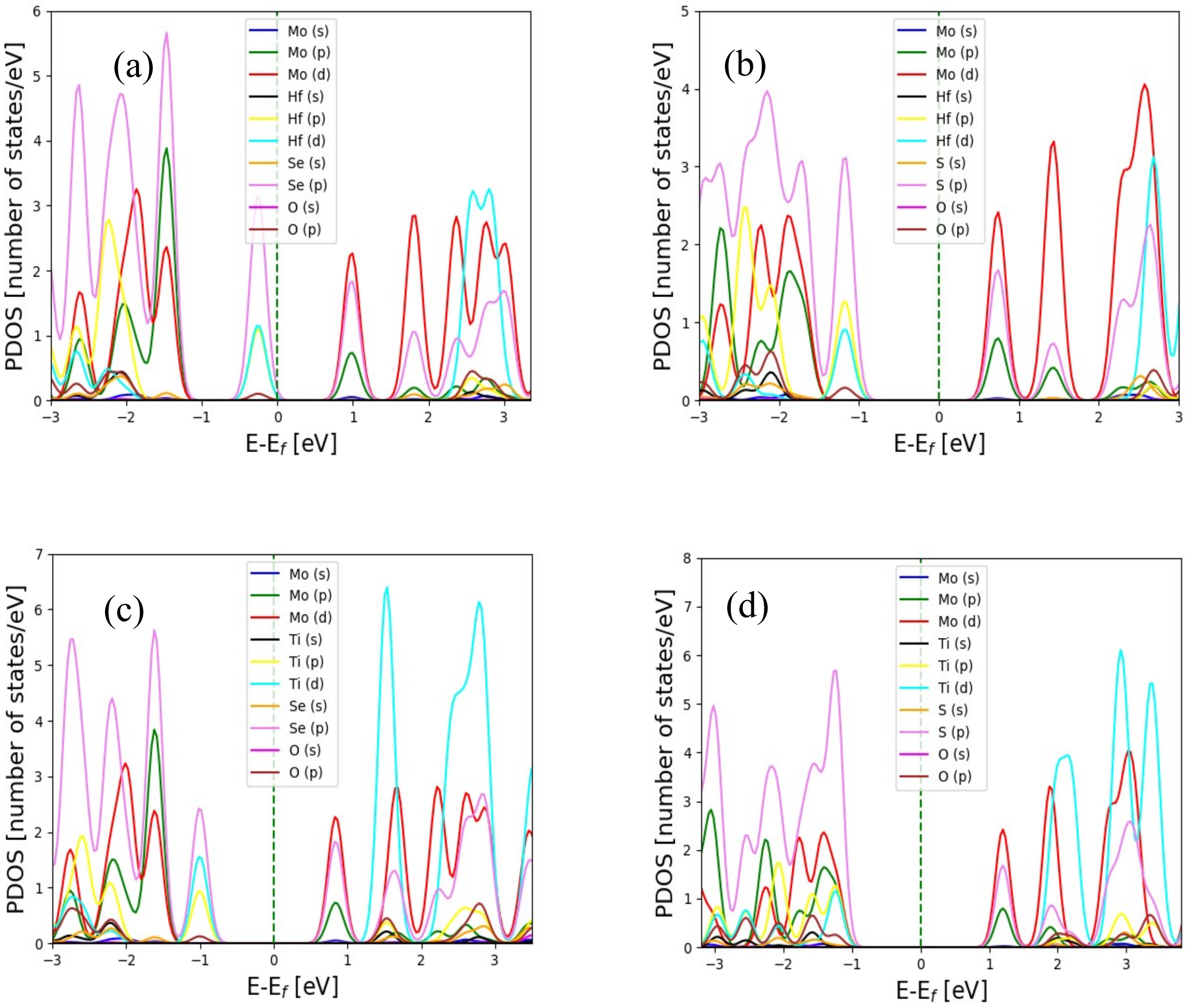

Figure S8: Bandgap of Distorted heterostructure using hybrid functional (a)T-HfSeO/MoSe2, (b)T-HfSO/MoS2, (c)T-TiSeO/MoSe2, and (d)T-TiSO/MoS2
